# Recurrent steroid-intolerant Hashimoto’s encephalopathy responsive to single-dose neonatal Fc receptor antagonist: a case report and literature review

**DOI:** 10.1186/s12883-026-04699-7

**Published:** 2026-02-23

**Authors:** Xiangliang Wang, Gang Wang, Jinjin Xu, Yongjie Bai, Wenjun Yan, Xinsheng Liu, Shukui Zhang, Ruile Shen

**Affiliations:** Department of Neurology, The First Affiliated Hospital, and College of Clinical Medicine of Henan University of Science and Technology, Luoyang, 471003 China

**Keywords:** Hashimoto’s encephalopathy, Efgartigimod-α, Neonatal fc receptor (FcRn) antagonists, Case report

## Abstract

**Background:**

Hashimoto’s encephalopathy (HE) is a rare, underdiagnosed, steroid-responsive autoimmune condition associated with thyroid autoimmunity.

**Case presentation:**

An 83-year-old female patient was admitted to hospital displaying symptoms of subacute cognitive decline, dysarthria and myoclonus. She had a history of Hashimoto’s thyroiditis and diabetes. Given the patient’s age and comorbid diabetes, the potential risks associated with steroid treatment were considered excessive. She was given an 800 mg dose of efgartigimod-α, which swiftly improved her ambulatory stability and cognitive function. Approximately four weeks later, the patient exhibited a clinical relapse and underwent a subsequent treatment cycle with efgartigimod-α. Each treatment cycle significantly alleviated the patient’s symptoms.

**Conclusions:**

The report under consideration herein highlights the clinical presentation, diagnostic challenges, and therapeutic considerations of HE, emphasizing the potential role of Neonatal Fc receptor (FcRn) antagonists, such as efgartigimod-α, as an emerging treatment strategy. The repeated administration of FcRn antagonists has been posited as a potential therapeutic intervention for patients diagnosed with hormone-intolerant Hashimoto’s encephalopathy.

## Background

 Hashimoto’s encephalopathy (HE) is an uncommon, potentially reversible neuropsychiatric syndrome associated with autoimmune thyroiditis. Since the seminal description by Brain et al. (1966), the construct has evolved, yet controversy persists regarding nosology, biomarkers, and optimal treatment [1–3]. Clinically, HE presents with fluctuating cognitive impairment, psychiatric symptoms, tremors or myoclonus, seizures, ataxia, and stroke-like episodes. Electroencephalogram (EEG) abnormalities are common but non-specific, and magnetic resonance imaging (MRI) findings are often normal or reveal non-specific white matter changes [2, 4, 5].

The exact pathophysiology of HE remains unclear, but it is believed to be related to autoantibodies targeting thyroid and neuronal tissues. Diagnostic criteria for HE typically include: (1) clinical presentation (e.g., cognitive impairment, myoclonus, seizures), (2) elevated thyroid autoantibodies, and (3) exclusion of other neurological or metabolic disorders. However, anti-thyroid peroxidase and anti-thyroglobulin antibodies may support the diagnosis but lack specificity, as they are also present in the general population and in other autoimmune disorders [3, 4]. Proposed autoantigens, such as the NH2-terminal of α-enolase, are promising but are not widely accessible and lack sufficient validation for routine clinical use [2].

Current expert frameworks increasingly position HE within the spectrum of autoimmune encephalitis (AE), emphasizing careful exclusion of mimics and judicious application of criteria for antibody‑negative AE [6, 7]. First‑line therapy has traditionally centered on corticosteroids, whereas rituximab or cyclophosphamide are used for escalation [6, 7]. However, prolonged steroid exposure is problematic in frail or diabetic patients, and a clinically significant minority shows relapse or steroid‑refractory disease [4, 5]. Emerging strategies that directly lower circulating Immunoglobulin G (IgG)—particularly the neonatal Fc receptor antagonist (FcRn) such as efgartigimod—offer a mechanism‑based option for IgG‑driven disorders. Efgartigimod accelerates IgG catabolism without affecting other immunoglobulins or albumin, has regulatory approval for generalized myasthenia gravis (MG), and has shown encouraging signals in antibody‑mediated AE [8–11]. Whether FcRn antagonist benefits patients labelled as HE remains an open question.

We present a case of recurrent HE responsive to efgartigimod-α and provide a comprehensive review of the literature regarding pathogenesis, diagnostic dilemmas, and therapeutic advancements, particularly focusing on novel immunotherapeutics.

## Case presentation

**Fig. 1:**
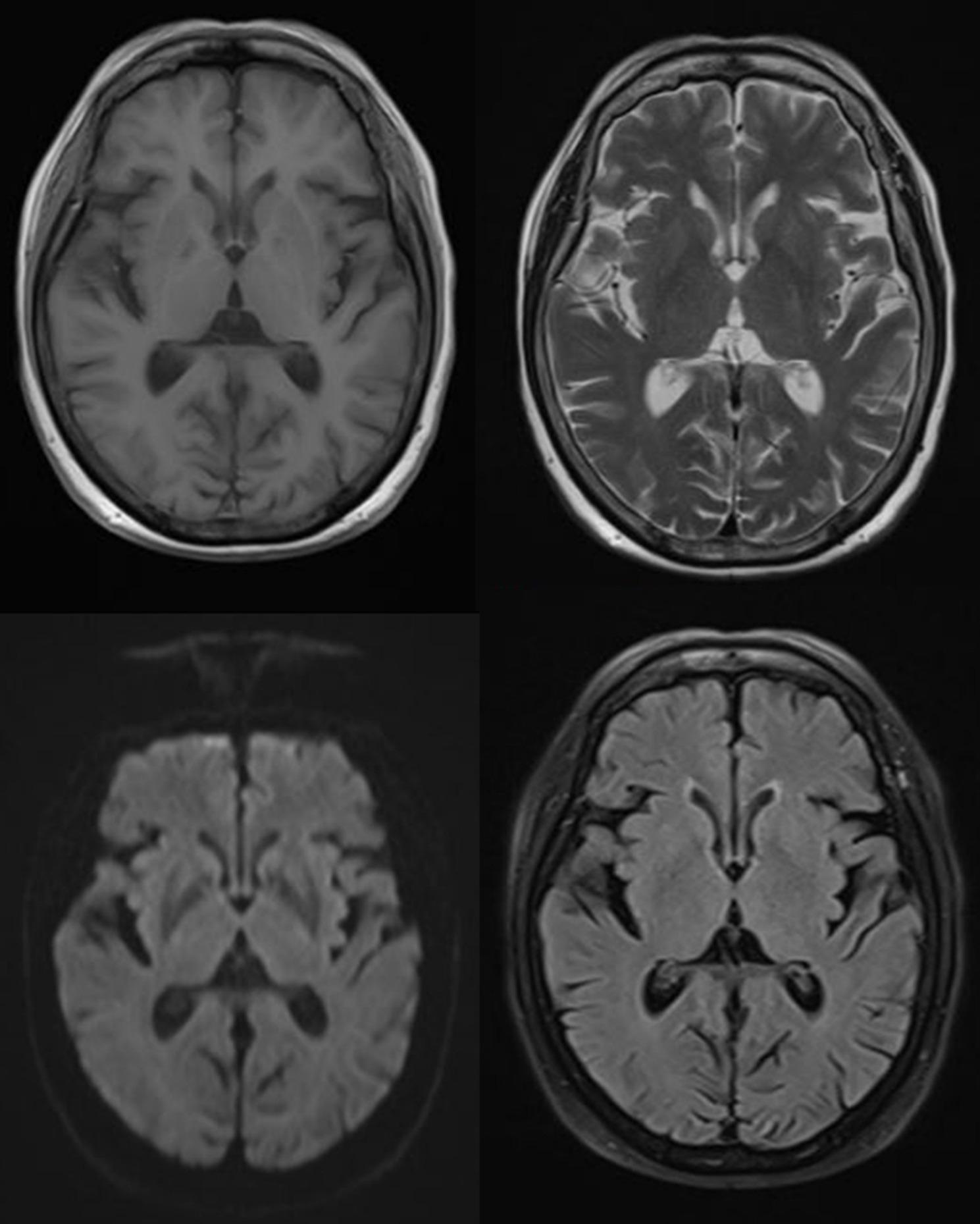
Neuroimaging findings showing T1WI, T2WI, DWI, and FLAIR sequences

An 83-year-old female patient presented with subacute gait unsteadiness and involuntary limb tremor/myoclonus that worsened with action and eased at rest, accompanied by dysarthria, memory decline, and cognitive slowing. Her medical history included autoimmune thyroiditis, managed with levothyroxine 50 µg daily, and type 2 diabetes mellitus. Neurological examination revealed that she was alert but disoriented, with an ataxic gait, generalized hyperreflexia, and stimulus-sensitive myoclonus. Baseline laboratory tests, rheumatologic panels, and tumor markers were unremarkable. Erythrocyte sedimentation rate was 8 mm/h. Free triiodothyronine and free thyroxine were both normal, but thyroid-stimulating hormone (8.300 mIU/L), anti-thyroid peroxidase antibody (299.9 IU/mL), and anti-thyroglobulin antibody (1775.0 IU/mL) were all elevated (Table 1). Thyroid ultrasound revealed a multinodular gland with diffuse parenchymal heterogeneity. No malignant features were observed in the cervical lymph nodes. Neuroimaging revealed bilateral periventricular white matter hyperintensities on T2-weighted imaging (T2WI) and FLAIR sequences. No significant abnormalities were observed on T1-weighted imaging (T1WI) or diffusion-weighted imaging (DWI). These findings were non-specific but consistent with previous descriptions of Hashimoto’s encephalopathy (Fig. 1). EEG revealed normal background activity without epileptiform discharges. Electromyography (EMG) findings demonstrated peripheral neuropathy in the lower limbs. Cerebrospinal fluid (CSF) analysis—including cell count, biochemistry, and cytology—showed markedly elevated protein levels in the absence of pleocytosis (Table 1). All comprehensive neurological autoantibody tests, including those for Langefier-related, ganglioside, anti-myelin-associated glycoprotein and autoimmune encephalitis-related antibodies, yielded negative results. Endocrinology consultation supported a diagnosis of Hashimoto’s thyroiditis with subclinical hypothyroidism. The thyroid surgeon proposed resection for thyroid nodules, but given age and preferences, surveillance was chosen. The patient was diagnosed with Hashimoto’s encephalopathy based on clinical features such as subacute encephalopathy, motor disorders and dysarthria, and elevated anti-thyroid antibodies. This diagnosis was made after excluding other pathogenic factors such as infectious encephalitis, metabolic encephalopathy and Creutzfeldt-Jakob disease. Given the patient’s age and comorbid diabetes, the potential risks associated with steroid treatment were considered excessive. Then she was treated with efgartigimod-α, a neonatal Fc receptor antagonist, at a dose of 800 mg via single intravenous infusion. The first dose resulted in a significant clinical improvement within 3 days, with gait stabilization, resolution of dysarthria, and reduction of myoclonus. Approximately four weeks later, the patient’s condition relapsed with symptoms similar to those experienced initially. The family opted for repeat treatment with efgartigimod-α. Within three days of administration, the patient’s symptoms showed significant improvement again. The family stated that the effect of the treatment lasts for about one month, after which the patient experiences a relapse. Each subsequent relapse exhibited the same symptoms as the initial relapse, and following each relapse, the patient received the same dose of efgartigimod-α treatment. The family reported that the patient’s symptoms were rapidly alleviated within three days of administration every time. Across episodes, no infusion reactions or serious infections occurred. Regretfully, due to the patient’s age, the family declined a repeat lumbar puncture for cerebrospinal fluid analysis. It is noteworthy that following the fourth treatment with efgartigimod-α, the family requested a modification to the treatment plan, citing their inability to accept the requisite tests prior to each efgartigimod-α administration and the associated financial burden. In light of the patient’s treatment requirements, a modification to their medication was deemed necessary, entailing the transition to azathioprine tablets at a daily dosage of 100 milligrams. During a telephone follow-up on November 2, 2025, the family stated that, following adjustments to the patient’s medication regimen, the patient had not experienced any further symptoms of Hashimoto’s encephalopathy, such as cognitive decline, dysarthria and myoclonus.

**Table 1:** Cerebrospinal fluid and biochemical investigations

Test	Result	Reference Range
CSF White blood cells (*10^9^ cells/L)	0.010	0–0.01
CSF Protein (mg/L)	1323.00↑	0.15–0.45
CSF Glucose (mmol/L)	4.94↑	2.8–4.5
CSF Oligoclonal bands (OCB)	Negative	Negative
Anti- thyroid peroxidase (U/mL)	299.9↑	0–34
Anti- thyroglobulin (IU/mL)	1775.0↑	0–115
Free triiodothyronine (pmol/L)	4.11	3.1–6.8
Free thyroxin (pmol/L)	12.80	12–22
Ultrasensitive thyroid-stimulating hormone(mIU/L)	8.300↑	0.27–4.2
Fasting blood glucose (mmol/L)	8.14↑	3.9–6.0

*10^9 ^cells/L represents 10 to the power of 9 (one billion) cells per liter of cerebrospinal fluid

The upward arrow (↑) indicates that the measured value is above the upper limit of the established reference range

## Discussion and conclusions

This case exemplifies the diagnostic and therapeutic challenges of HE in the elderly. Several features increased diagnostic confidence despite the absence of neuronal surface antibodies: subacute encephalopathy with movement disorder and dysarthria; elevated anti-thyroid antibodies; non-specific EEG/MRI; bland CSF with markedly protein elevation; exclusion of infectious, structural, metabolic, and neoplastic mimics; and a robust, reproducible response to immunotherapy [2–5, 7].

The specificity of anti-thyroid peroxidase and anti-thyroglobulin antibodies for HE is limited, as titers correlate poorly with disease activity and are frequently positive in euthyroid individuals [3–5]. Antibodies against the NH2‑terminal of α‑enolase may represent an autoimmune endophenotype, but their clinical utility remains exploratory. Contemporary frameworks recommend classifying such cases as probable antibody-negative autoimmune encephalitis (AE), provided that alternative diagnoses are rigorously excluded and supportive inflammatory findings (EEG/MRI/CSF) are demonstrated where possible [6, 7]. Our patient satisfied pragmatic criteria for probable antibody negative AE overlapping with the historical HE concept, reinforcing the need to harmonize terminology across neurology and endocrinology.

FcRn salvages IgG from lysosomal degradation, extending its half-life. Efgartigimod, a human IgG1-derived Fc fragment, competitively inhibits FcRn and accelerates systemic IgG clearance, without affecting IgA, IgM, or albumin levels [8, 10, 11]. In generalized MG, efgartigimod has been shown to improve activities of daily living and quantitative MG scores, while maintaining a favorable safety profile. Emerging data also suggest efficacy in other IgG-mediated disorders. In AE, growing-albeit early-evidence suggests that efgartigimod rapidly improves clinical symptoms, reduces serum/CSF IgG and neuronal antibody titers, and demonstrates an acceptable safety in anti N-methyl-D-aspartate receptor and ‌leucine-rich glioma inactivated 1 encephalitis cohorts [8, 9, 12]. Our observation of temporally consistent remission following each single 800 mg infusion, with relapse occurring around four weeks later, aligns with the pharmacodynamic profile of IgG re-accumulation after FcRn antagonists reported in other IgG-mediated disorders [11].

This case highlights the potential utility of efgartigimod-α (a neonatal Fc receptor antagonist) as a treatment option for HE, particularly in patients who cannot tolerate steroid treatments due to age and comorbid conditions such as diabetes. The significant improvement in both cognitive function and gait stability following a single dose of efgartigimod-α demonstrates its efficacy in steroid-sparing treatment strategies, providing novel evidence for its role in treating HE in such patients. Given the lack of established therapeutic options for these patients, this report offers valuable insight into potential alternatives. In elderly or diabetic patients with HE who are intolerant to corticosteroids or experience relapses, FcRn inhibition may be considered as part of a step-up treatment strategy following standard first-line therapies-including corticosteroids, intravenous immunoglobulin (IVIG), and plasma exchange-and malignancy screening [6, 7].

However, as a single-case report, the findings of this study are limited in their generalizability. While the patient’s improvement suggests the potential efficacy of efgartigimod-α in HE treatment, larger, controlled studies are needed to validate its broader use. The short follow-up period and the recurrence of symptoms highlight the need for further investigation into the long-term efficacy and potential maintenance treatment strategies. Additionally, the patient’s condition could have been influenced by unaccounted factors, such as underlying metabolic changes or the progression of autoimmune thyroid disease, which require further exploration. The absence of histopathological evidence, such as brain biopsy or other definitive diagnostic techniques, also limits our ability to confirm the exact pathological mechanism of HE in this patient.

In summary, this case emphasizes that non-steroidal treatments, such as FcRn antagonists, represent a promising alternative for managing HE, particularly in patients with contraindications to steroids. However, while this report introduces a potentially effective therapeutic option, it also underscores the necessity for further research to evaluate the long-term safety and sustained effectiveness of efgartigimod-α in the treatment of HE.

## Data Availability

All data generated or analyzed during this study are included in this published article.

## Abbreviations

HE: Hashimoto’s encephalopathy
FcRn: Neonatal Fc receptor
EEG: Electroencephalogram
MRI: Magnetic resonance imaging
AE: Autoimmune encephalitis
IgG: Immunoglobulin G
MG: Myasthenia gravis
EMG: Electromyography
CSF: Cerebrospinal fluid
